# Reducing the risk of sudden unexpected infant death: the caffeine hypothesis

**DOI:** 10.1038/s41372-025-02333-x

**Published:** 2025-06-10

**Authors:** Thomas Hegyi, Barbara M. Ostfeld

**Affiliations:** 1 The Division of Neonatology, Department of Pediatrics, Robert Wood Johnson Medical School, Rutgers, The State University of New Jersey, New Brunswick, NJ USA; 2 The SIDS Center of New Jersey, Robert Wood Johnson Medical School, Rutgers, the State University of New Jersey, New Brunswick, NJ USA

**Keywords:** Epidemiology, Risk factors

## Abstract

This review proposes that intermittent hypoxia is the primary pathogenic mechanism driving Sudden Infant Death Syndrome (SIDS). Intermittent hypoxia is a powerful source of molecular and cellular injury and is frequently experienced by infants, especially under conditions associated with known SIDS risk factors such as prone sleeping, respiratory infections, and prenatal nicotine exposure. These factors often trigger hypoxic episodes that may impair autonomic regulation, hinder arousal from sleep, and damage critical neural circuits. By integrating current data, this review highlights the central role of intermittent hypoxia in SIDS pathophysiology. Additionally, it evaluates the potential of caffeine, a respiratory stimulant and adenosine receptor antagonist, as a protective intervention to reduce SIDS risk by enhancing respiratory stability and arousal capacity.

## Introduction

Sudden Unexpected Infant Death (SUID) is the sudden death of an infant, whether explained or not, as defined by the National Center for Health Statistics. This definition encompasses deaths attributed to Accidental Suffocation and Strangulation in Bed, Ill-defined and Unknown Causes, and Sudden Infant Death Syndrome (SIDS). SIDS is a death that remains unexplained even after an investigation that includes an autopsy and a review of the death scene and clinical history [1]. According to the Centers for Disease Control and Prevention, in the USA, SIDS is the leading cause of death among infants aged 1–12 months, and it is the third leading cause of all infant deaths from birth to 12 months of age. In 2022, there were 1529 deaths from SIDS, representing 41% of the three SUID subcategories that year.

SIDS has been an enduring human affliction; some even say it is biblically referenced during the time of King Solomon. It has been attributed to a multitude of causes varying from status thymico-lymphaticus in 1890 to a brainstem abnormality in 1983. In 1987, Hunt and Brouillette [2] declared that “the most compelling hypothesis continues to be that SIDS is related to a brainstem abnormality in the neuroregulation of cardiorespiratory control” a statement that is supported by research showing that the brainstem plays a critical role in respiratory and autonomic regulation, sleep, and arousal and that defects in brainstem function lead to impaired autoresuscitation, abnormal respiratory patterning, obstructive apnea during sleep, autonomic dysfunction, and arousal deficits [3–13].

Despite the recognized genetic predisposition and familial occurrence of SIDS, in 1994, Filiano and Kinney proposed a model for SIDS pathogenesis: the simultaneous existence of infant vulnerability during a critical developmental period in the presence of an exogenous stressor [14]. The adoption of this model was validated by a reduction in the SIDS/SUID rate, which emphasized the elimination of stressors. Animal studies have confirmed that these factors affect apneas, autoresuscitation, and arousal, and have identified molecular mechanisms, neurotransmitters, and specific brain pathways explaining the observations [15]. Key insights from these investigations have shown that infants who die of SIDS have a high propensity for respiratory inhibition by hypoxia combined with a reduced capacity to auto-resuscitate and arouse to recover from these events. In 2009, Kinney proposed the idea that a subset of SIDS cases stemmed from a disorder of the medullary neurotransmitter serotonin and related neurotransmitter systems that impair responses to stressors during sleep [16]. These observations suggested that the final pathway to SIDS involved a combination of immature cardiorespiratory control and a failure of arousal from sleep [17]. Other abnormalities have been identified that could impair autonomic cardiorespiratory control, including deficiencies in orexin [18], abnormalities of substance P and neurokinin 1 receptor [19], and reduced levels of butyrylcholinesterase, an enzyme in the cholinergic system [18].

While the Triple Risk Model may provide a valuable explanation for SIDS pathophysiology, it lacks a precise single pathological mechanism. Most risk factors that define vulnerable infants share a common theme: intermittent hypoxia (IH), characterized by a drop in oxygen saturation below 80% for at least five seconds [19]. These risk factors include high-risk pregnancies identified by anemia, poor weight gain, intrauterine growth retardation, maternal smoking, and prematurity [20, 21]. Infant arousal has been shown to be compromised by nicotine exposure and opiate exposure [22–24]. The presence of chronic IH prior to SIDS has been confirmed by markers of tissue hypoxia, including hyperplasia of pulmonary neuroendocrine cells [25], reduced vitreous humor hypoxanthine oxidase [26], elevated vascular endothelial growth factor in the cerebrospinal fluid [27], nonspecific gliosis in the brainstem [28, 29] and brainstem nuclei apoptosis, consistent with hypoxia-induced programmed cell death [30, 31]. Postnatal growth retardation, elevated serum cortisol, and fetal hemoglobin levels also support this conclusion. The environmental factors that act as stressors in this model include prone sleeping position [32], head covering, bed sharing [33], maternal smoking [34, 35], and high altitude [36], all of which can lead to hypoventilation and IH [37]. Interventions that increase ventilation, such as a fan to ensure better room air circulation, reduce SIDS incidence [38]. Pacifier use, which enhances airway patency, is another protective factor [38].

## Neonatal hypoxia

Neonatal hypoxia has long been considered a mechanism behind SIDS [39]. During development, hypoxia prevents the normal expression of genes encoding ion channels and may increase the risk of arrhythmogenic death [39, 40]. Mice born into a hypoxic environment or exposed to increased myocardial hypoxia exhibit delayed electrocardiac maturation and significantly higher rates of sudden death [41]. Another theory posits that hypoxia can reduce diaphragm force-generating ability and increase diaphragm workload, leading to diaphragm weakness and respiratory failure [42].

The preterm infant’s response to a hypoxic challenge is uniquely characterized by paradoxical hypoventilation, a response that has recently been found to extend beyond the neonatal period [43]. This phenomenon is more prevalent in males [44], reflecting the increased rate of SIDS among this group. A delay in the transition from this immature ventilatory response should be considered in the context of sudden unexpected postnatal collapse [45].

Autopsy findings after SIDS often show evidence of IH, and include intrathoracic petechiae, dilated right ventricle, increased hepatic erythropoiesis, thickening of the smooth muscle walls of the small pulmonary arteries, and gliosis of the brain stem in areas of respiratory control [46]. Central and peripheral chemoreceptors undergo critical development during the initial postnatal period, consistent with the age range of SIDS [47]. Examination of the carotid body in SIDS victims has demonstrated reductions in glomic tissue volume and cytoplasmic granules of type I cells, changes in cytological composition, and increases in dopamine and noradrenaline contents. Prematurity, exposure to tobacco smoke, and intermittent hypoxia are among the factors known to adversely affect carotid body functional and structural maturation [48].

## Intermittent hypoxia

IH is a common finding in preterm infants. The fetus responds to hypoxemia with decreased breathing activity caused by central respiratory ventilatory depression, which results from descending pontine or suprapontine inhibition [49]. In preterm infants, immature respiratory control manifests as apnea, with increasing gestational age reducing the vulnerability to these episodes. IH episodes decrease with advancing postnatal age [49] and may have transient beneficial effects, such as enhanced chemoreceptor sensitivity, improved respiratory control, and enhanced adaptive response [19]. However, these events are associated with multiple adverse outcomes, including retinopathy of prematurity, bronchopulmonary dysplasia, sleep-disordered breathing, neurodevelopmental handicap, and death [49]. IH can further compromise the infant by causing respiratory instability during sleep and by blunting the ventilatory response to acute hypoxia [50].

The mechanisms of injury caused by IH are the focus of several investigations. Evidence from animal models indicates that IH leads to changes in inflammatory signaling and the generation of reactive oxygen species [51, 52]. IH may alter neuronal migration by reducing reelin expression in the developing piglet hippocampus [53] and may cause white matter hypomyelination through a mechanism that differs from the loss of myelin-producing cells and axons occurring in periventricular leukomalacia [54].

We propose that intermittent hypoventilation and IH, when uninterrupted by arousal or compensatory mechanisms, initiate a self-reinforcing cascade of physiological decline, culminating in escalating asphyxia, profound bradycardia, worsening metabolic acidosis, severe hypoxemia-induced gasping, and ultimately, death.

## Caffeine

The causal relationship between IH and SIDS has not been addressed, and there is no evidence from prospective investigations. However, indirect evidence suggests that IH may contribute to some cases of SIDS. Caffeine is utilized to treat apnea and has been demonstrated to reduce IH [55–57]. Caffeine administration has been shown to preserve white matter development and myelination while improving neurological outcomes in premature human infants and neonatal rats exposed to chronic hypoxia [58, 59]. In the 5-HT deficient animal model, caffeine accelerated the onset of gasping and improved autoresuscitation after a brief period of asphyxia insult in a dose-dependent manner [60]. The subsequent recovery of heart rate and restoration of breathing led to improved survival. Caffeine-related improvements in ventilation and oxygenation prevent the onset of hypoxia and the irreversible cascade toward cardiac arrhythmia [39]. With increasing evidence that the 5-HT system in the brainstem is essential for successful autoresuscitation, Cummings and coworkers demonstrated that administering caffeine to 5-HT-deficient mice improved the time to the onset of gasping, recovery of breathing, and restoration of heart rate in a dose-dependent manner [61]. Fernstrom has shown that caffeine injection elevates brain 5-HT levels [62] and increases cortical 5-HT receptors [63].

The metabolism of caffeine has been extensively studied in newborn infants. A consistent observation is that serum caffeine concentrations are variable, with changes occurring as infants mature [64]. This variability may, in part, be attributed to a change in the apparent volume of distribution, but this remains constant (~0.8–0.9 L/kg) with advancing postnatal age [65]. Caffeine’s half-life decreases with maturity. Aldridge found that during the first month of life, caffeine accounted for more than 85% of the identifiable products in urine [66]. Caffeine remained the predominant component for the first 3 months, but its percentage gradually decreased to the adult value of less than 2% by the age of 7–9 months. The different pathways of caffeine metabolism also vary with postnatal age [67]. Total demethylation and N3- and N7-demethylation increase exponentially with postnatal age, reaching a plateau by 120 days. N1-demethylation shows no variation with postnatal age, suggesting that N3-demethylation is more critical in young infants and that maturation of N1-demethylation occurs later than 19 months of age. 8-Hydroxylation matures as early as 1 month of age and may be higher in infants than in adults. Acetylation is not mature before at least 1 year of age, but differences in the maturation rate of acetylation may be related in part to genetic acetylation status [67]. With caffeine levels remaining in the infant for a prolonged yet variable length of time, it is noteworthy that the risk of SIDS is delayed, peaking between two and four months of age [68]. This observation has evaded explanation until now, but it is important to note that the prevalence of caffeine consumption is almost universal in pregnancy [69].

Caffeine, especially in beverages, is widely consumed in the USA in various forms and by people of all ages [70]. Frary and colleagues noted that 89% of women between 18 and 34 years of age consumed caffeine with an average intake of 166 mg/kg/day [71]. However, the percentage and amount of caffeine consumed by pregnant women appear to have decreased. Surveys indicate that 68–74% of pregnant women consumed caffeine. Pregnant caffeine consumers had an average intake ranging between 125 mg/day and 193 mg/day [72].

Following ingestion, caffeine is fully absorbed from the gastrointestinal tract, with a mean half-life of approximately four hours in healthy adults [73]. Stavshansky examined the pharmacokinetics of caffeine in breast milk in 1988 [74]. Six healthy lactating women received 100 mg of caffeine, and plasma and breast milk levels were measured by using gas-liquid chromatography. Caffeine was rapidly absorbed, with peak times ranging from 0.50 to 1.00 h, and peak levels between 3.60 and 6.15 μg/ml. In breast milk, the time to peak ranged from 0.75 to 2.00 h, and levels ranged from 1.98 to 4.30 μg/ml. The breast milk/plasma ratio was approximately 0.8. In another investigation, Tyrala reported that peak concentrations in breast milk were achieved one hour after the mother ingested caffeine [75]. Therefore, in these studies, caffeine rapidly entered breast milk and may be administered to the infant via this vehicle.

The effects of maternal caffeine intake during pregnancy and lactation have been studied in animal models. Lorenzo noted that caffeine intake during these periods significantly reduced adenosine receptors in male but not in female offspring [76]. In another investigation, Chapman observed that caffeine attenuated a decrease in ventilation stimulated by moderate alveolar hypoxia [77]. It is crucial to examine caffeine levels in different ethnic populations due to the presence of genetic variants that lead to varying rates of caffeine metabolism. The mechanism of this variability involves higher N-acetyl transferase activity, lower xanthine oxidase activity, and lower CYP1A2 activity in African Americans compared to Caucasians [78].

## SIDS prevention

Prevention of SIDS has focused on eliminating risk factors and encouraging protective behaviors such as breastfeeding [79]. A recent meta-analysis showed that breastfed infants were less likely to die from SIDS compared to bottle-fed infants [80]. However, the mechanism of this protection, which has not been confirmed in all studies, has not been identified. We hypothesize that the protection afforded by breast milk [80] is due to caffeine, which could be provided to the infant through breastfeeding. This proposal is not supported by a study by Ford and coworkers, who found that infants of mothers with heavy caffeine intake during pregnancy had an increased risk for SIDS [81]. However, a closer examination of the findings reveals that it was the withdrawal of caffeine that increased the risk. The authors proposed that caffeine altered the fetal respiratory center, and its withdrawal left the infant with an inadequate respiratory drive. An alternate explanation, again based on caffeine withdrawal, was that caffeine exposure in utero increased the vulnerability of the infant to hypoxia.

Most SIDS cases occur during the first six months of life, and it is critical to show that caffeine concentrations in breast milk can be maintained within a narrow range by its administration. Administration of 100 mg of caffeine to six lactating women resulted in peak breast milk levels from 0.75 to 2.0 h, with levels ranging between 1.98 and 4.3 μg/ml, reflecting ~80% of plasma concentrations [74]. Ryu determined the caffeine concentration in the breast milk of nine lactating women ingesting 750 mg of caffeine daily and the concentration of caffeine in their infants [82]. The caffeine levels in breast milk averaged 4.3 μg/ml, ranging between less than 0.25 μg/ml and 15.7 μg/ml. The concentration of caffeine in the infants’ sera was 1.4 μg/ml (range: nondetectable to 2.8 μg/ml). While maternal caffeine ingestion occurred only during the first five days of the study, caffeine was still detectable in infants’ sera at nine days.

While caffeine levels in infants are variable and based on diet, it is essential to ensure infant safety. Maternal caffeine consumption during gestation affects hematologic parameters in both rat and human infants and induces long-term effects on sleep, locomotion, learning abilities, and anxiety in rodent offspring; however, there is no evidence of these effects in humans. However, while risks for the newborn infant associated with caffeine during pregnancy include smaller birth size, obesity [83], and impact on brain development, the risks associated with caffeine during breastfeeding have been limited to irritability, fussiness, and sleep disturbances. Caffeine does not change breast milk composition but instead stimulates milk production. Its consumption during gestation and lactation has no measurable consequences on the fetus and the newborn infant [84].

Clearly, studies examining the relationship between caffeine, breast milk, and SUID must continue before considering any recommendations for introducing caffeine in the context of SIDS reduction. Investigations should assess its efficacy and safety, the feasibility of its use, and its interaction with other risk factors, such as smoking. Indicators need to be established to help clinicians identify the infants who would benefit most from this preventive approach. Finally, the potential adverse effects of caffeine intake in healthy women must be weighed against the benefits for SIDS risk reduction in the infant [85].
